# Evaluation of Cyclooxygenase-2 Expression in Association with Clinical-Pathological Factors in Malignant Melanoma

**DOI:** 10.30699/IJP.14.2.96

**Published:** 2019-01-10

**Authors:** Amir Hossein Jafarian, Nema Mohamadian Roshan, Masoumeh Gharib, Vahid Moshirahmadi, Aida Tasbandi, Amir Ali Ayatollahi, Hossein Ayatollahi

**Affiliations:** 1 *Associate Professor of Pathology, Department Of Pathology, Cancer Molecular Pathology Research Center, Faculty of Medicine, Mashhad University of Medical Sciences, Mashhad, Iran*; 2 *Assistant Professor of Pathology, Department Of Pathology, Cancer Molecular Pathology Research Center, Faculty of Medicine, Mashhad University of Medical Sciences, Mashhad, Iran*; 3 *Cancer Molecular Pathology Research Center, Faculty of Medicine, Mashhad University of Medical Sciences, Mashhad, Iran*

**Keywords:** Malignant melanoma, COX-2, Prognostic Factors

## Abstract

**Background and Objective:**

The primary goal of this study is to develop a rigorous understanding of the correlation between COX-2 expression and malignant melanoma prognostic factors.

**Material and Methods:**

In this cross-sectional study, we analyzed 60 cases of cutaneous malignant melanoma. The related stained slides were reviewed by two pathologists. The results were interpreted according to the COX2 staining index (SI), tumor thickness (Breslow, Clark), number of mitoses per 10 hpf, and melanoma types. Gender, lymph node involvement, metastasis, and survival were considered as evaluation factors as well.

**Results:**

The expression of the COX-2 protein was evident in 98.4% of cases. A strong Staining Index(SI) was reported in 60% of all melanomas, moderate staining was detected in 20.8% and weak staining in 10%; 1.6% of studied cases showed no staining. Benign nevus specimens showed no staining for the COX-2 enzyme.

**Conclusion:**

We have demonstrated that COX-2 is strongly expressed in the majority of malignant melanomas and that the SI score of COX-2 is related to the number of mitoses, tumor thickness (based on Clark level and Breslow), melanoma sub-type, lymph node involvement, and metastases; No association was noted between the anatomic site, gender, and survival. COX-2 can be applied as a prognostic factor in malignant melanoma and a promising candidate for future target therapies.

## Introduction

Malignant melanoma is the third most common and serious form of skin cancer, (1) with 75% mortality rate and an annual global incidence of 160,000 (2); these numbers are believed to double every 10 to 20 years (3). It has been noted that it is more prevalent among the elderly and is one of the most common lethal cancers in young adults (aged 15-39 years) with a 20% chance of metastases leading to death (4). Due to the rising incidence and highly distant metastatic preference, as well as chemoprophylactic unresponsiveness and poor prognosis in its late stage (a five-year survival rate of 16 % for metastatic melanoma), early-stage diagnosis and preventive interventions may reduce its high mortality rate (4-7). Thin lesions with metastases have also been reported in several studies, which render the prognosis unreliable at early stages, and indicate the necessary need for new and more precise prognostic biomarkers (8). Immunohistochemistry is used for diagnostic evaluation when the diagnosis of early melanoma is difficult (9). Today, it is also applicable for metastatic tumors of unknown origin. Surface receptors involved in cell adhesion and cell cycle regulator genes, or gene products, are also being widely evaluated as biological markers (10-14). In the few studies conducted in Iran, an estimated rate of 0.3 new cases per 100,000 annually, has been reported (4).

In a study conducted by Noorbala et al. during 1988 to 2008 in Yazd, a warm province in the center of Iran, the mean incidence rate for cutaneous malignant melanoma (CMM) was 0.40 per 100,000 for males and 0.27 per 100,000 for females yearly (15). Due to previously mentioned reasons, many studies have focused on Cyclooxygenase‐2 (COX -2) as an inducible enzyme that is activated by UV radiation, and is a known risk factor for melanoma development (16-18). The generation of prostaglandins through COX-2 catalysis appears to be responsible for the proliferation, invasiveness and metastatic characteristics of the tumor. COX-2 is also expressed in various tumor types, and expression levels are associated with invasiveness and prognosis in some tumor entities; this suggests an important role for COX-2 in tumor development and progression (19). According to a limited number of studies on COX2 expression in melanoma in our region, the aim of this study is to assess correlations between COX-2 expression and the clinical-pathologic prognostic characteristics of primary melanoma. 

## Materials and Methods

**Patients and tissue samples**

Seventy-One Formalin-Fixed Paraffin-Embedded (FFPE) tissues were obtained from the archives of the pathology departments of the Ghaem and Omid hospitals, located in Mashhad, Iran. This study was approved by the Research Deputyship of the Mashhad University of Medical Sciences in terms of ethical and methodological issues. According to the exclusion criteria, which are elaborated below, 11 cases were omitted from the study. Therefore, 60 cases of malignant cutaneous melanomas representing different stages and grades of tumor progression were analyzed regarding histopathological and immunohistochemical changes. The samples were selected from those patients who underwent punch, incisional & excisional skin biopsies during 2002-2008 at cited centers. The tissues had been fixed in 10% formalin and embedded in paraffin, routinely. The related Hematoxylin and Eosin-stained slides were reviewed by two pathologists. After that, we determined tumor thickness based on Clark and Breslow criteria, and then appropriate zones were located on slides for immunohistochemistry. Formalin-fixed paraffin blocks with 3 microns of thickness were cut and transferred into a microwave, using the EDTA buffer. After several steps of washing with the buffer (TBS), the slides were covered with primary monoclonal COX-2 antibodies (Cat No: M3617, Dako, Denmark) and placed in a moist chamber. In the next step, the tissues’ surfaces were coated with secondary antibodies and a peroxidase-labeled polymer (K4003, Dako, Denmark). Finally, tracking was done and observed using EnVision, Dako Envision ^TM^ (DakoCytomation Protocol), and staining. We selected 60 formalin-fixed paraffin embedded tissue samples taken from various patients. A non-tumoral normal colon was used as a positive control, while a sample that underwent the elimination of primary antibodies was used as the negative control. Also, 10 formalin-fixed paraffin embedded samples with benign nevi were stained with COX-2, as well as normal skin tissue; they were used for comparison with 60 tumoral samples and to verify whether the COX-2 staining is observed. 

**Inclusion criteria:**

The criteria to enter a sample in this study were the availability of information on patients’ age and gender, as well as an unequivocal histological diagnosis in related files. The presence of enough tissue for the procedure was also necessary. 

**Exclusion criteria**

Incomplete patient records (file content), inadequate tissue, and the absence of paraffin blocks were the parameters which excluded a sample from the study. 

**Interpretation of results:**

Stained slides with COX-2 were reviewed by two expert pathologists with an optical microscope (Nikon, made in Japan, 100 x and 400x). Then, a scoring system from 0 to 3+ (staining index: SI score) was applied. The results were interpreted according to the COX-2 staining score (SI), tumor thickness (Breslow, Clark), mitoses number per 10 hpf and melanoma types. Gender, lymph node involvement, metastasis, and survival were considered as evaluation factors as well. 

**COX-2 scoring system:**

The intensity of COX-2 staining was denoted by the COX-2 (SI) scoring system. The cytoplasmic intensity of COX-2 staining was specified for each specimen on a scale of 0–3 as follows (23): 

0= negative (no staining of melanoma; staining of epidermal or endothelial cells is observed, or staining of melanoma cells is less than 30% of tumor cells); 

1= weak positive staining (staining is at least equal to that of epidermal or endothelial cells and is detected in at least 30% of melanoma cells); 

2= moderate positive staining (staining is more extensive than epidermal or endothelial cells and is detected in at least 30% of melanoma cells); 

3= strong positive staining (staining is obviously stronger than that of epidermal or endothelial cells and is detected in at least 30% of melanoma cells).

**Statistical Analysis:**

We used parametric (chi squared & independent T-test) and non-parametric tests 

(Mann-Whitney test) for data analysis and interpretation, depending on the type of variables. We also tried to use logistic regression for data analysis, but it was impossible due to the low number of samples and variable diversity.

## Results

**Figure 1:**
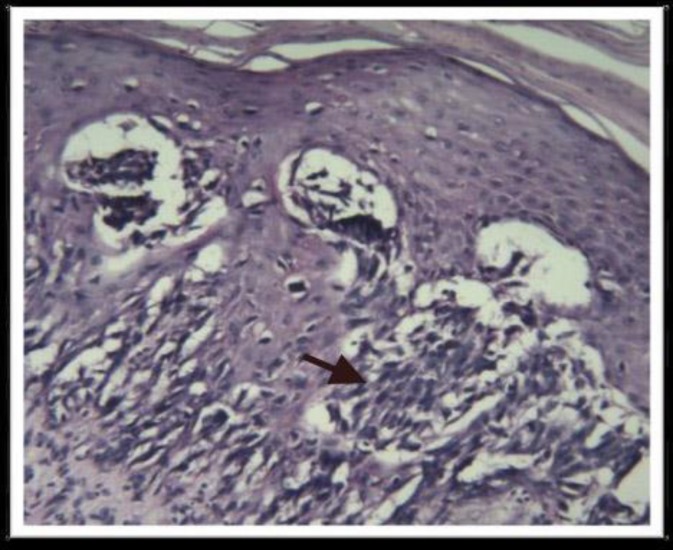
A case of superficial spreading melanoma with H&E staining at X400 magnification

**Figure 2:**
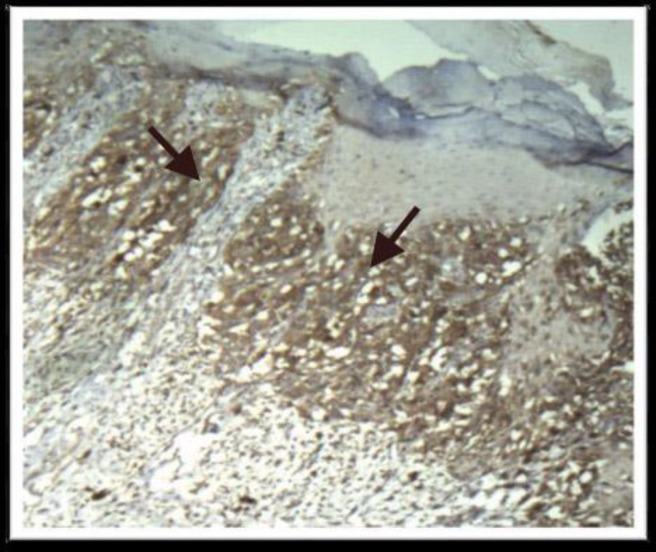
Severe staining for COX-2

In this study, 60 patients (27 males and 33 females) with melanocytic skin lesions were examined. Considering the level of involvement, based on Clark levels, for malignant melanoma, there were 3 cases (5 %) with level I (Intra-epidermal; *in situ*) )Figure 1), 8 cases (13.3 %) with level II (papillary dermis invasion), 17 cases (28.3%) with level III (invasion up to the surface of the reticular dermis), 27 cases (45%) with level IV (deep reticular dermis invasion) and 5 cases (8.3%) with level V (invasion up to the subcutaneous fat). Strong staining was found in 60% (36 of 60) of studied melanomas (Figure 2); moderate staining was detected in 26.8% (16 of 60), weak staining in 11.6% (8 of 60), and only one melanoma (lentigo maligna melanoma) showed no staining. In addition, benign nevus specimens were analyzed, for comparison with malignant melanoma, and showed no staining for COX-2.

It is known that COX-2 is strongly expressed in the majority of all melanomas. 

**Figure 3:**
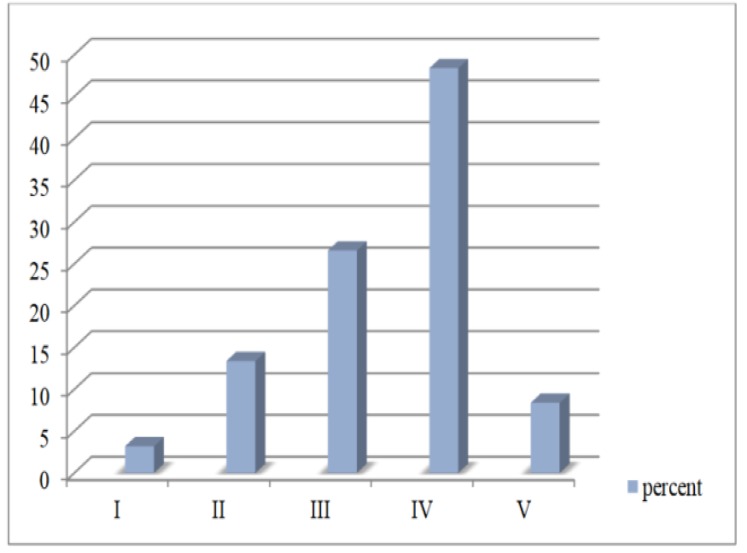
COX-2 expression in different Clark levels

Malignant melanoma tumor thicknesses were measured under a microscope. The Breslow thickness was less than 0.76 mm in 3 cases (5%), 0.76-1.5 mm in 5 cases (41.7%), and above 1.5 mm in 32 cases (53.3%). As for the Clark level, the utmost expression of COX-2 was found at greater Clark levels and was statistically significant (*P*<0.001). COX-2 expression levels in different Clark levels are demonstrated in Figure 3.

**Figure 4:**
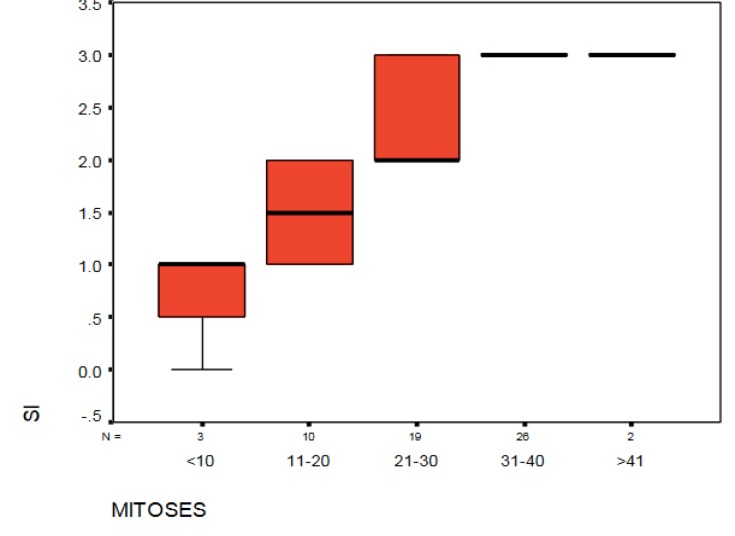
The relationship between the SI score and mitoses

From the data in Figure 4, it is apparent that there was a significant association between the SI score and the number of mitoses (*P*<0.001).

A meaningful correlation between melanoma subtypes and the SI score (*P*<0.01) is highlighted in Table 1.

The results of parametric tests (Chi-squared and independent T-test) which are depicted in Table 2, indicate a significant relationship between tumor thickness (based on Breslow criteria) and the SI score. (*P*<0.001)

We analyzed the correlation between lymph node involvement and the SI score; as seen in Table 3, a significant relationship was found between cited parameters (*P*<0.001).

As illustrated in Table 4, the results of the non-parametric tests (Mann-Whitney test) provide a significant relationship between metastases and the SI score (*P*<0.001).

No correlations were observed between COX-2 expression and overall survival in our evaluated individuals regarding the Kaplan-Meier curves.

**Table1:** The Relationship between SI score and types of melanoma

Types of melanoma	Count	Negative (0)	Mild (1+)	Moderate (2+)	Severe (3+)	P-value
Acral	9	0	1	4	4	<0.001
Nodular	35	0	0	3	32	
Superficial	6	0	3	3	0	
Lentigo maligna	4	1	2	1	0	
Other	6	0	1	5	0	
Total	60	1	7	16	36	

**Table 2:** Tumor thickness and SI score relationship

Breslow(mm)	Count	Negative (0)	Mild (1+)	Moderate (2+)	Severe (3+)	P-value
**< 0.76**	3	1	2	0	0	<0.001
**0.76-1.5**	25	0	5	15	5	
**>1.5**	32	0	0	0	32	
**Total**	60	1	7	15	37	

**Table 3:** The Relationship between SI score and Lymph node involvement

Lymph node involvement	Count	Negative (0)	Mild (1+)	Moderate(2+)	Severe (3+)	P-value
**Present**	22	0	0	2	20	<0.001
**Absent**	38	1	7	13	17	
**Total**	60	1	7	15	37	

**Table 4:** The Relationship between SI score and metastases

Metastasis	Count	Negative (0)	Mild (1+)	Moderate(2+)	Severe (3+)	P-value
**Present**	23	0	0	2	21	<0.001
**Absent**	37	1	7	13	16	
**Total**	60	1	7	15	37	

**Figure 5:**
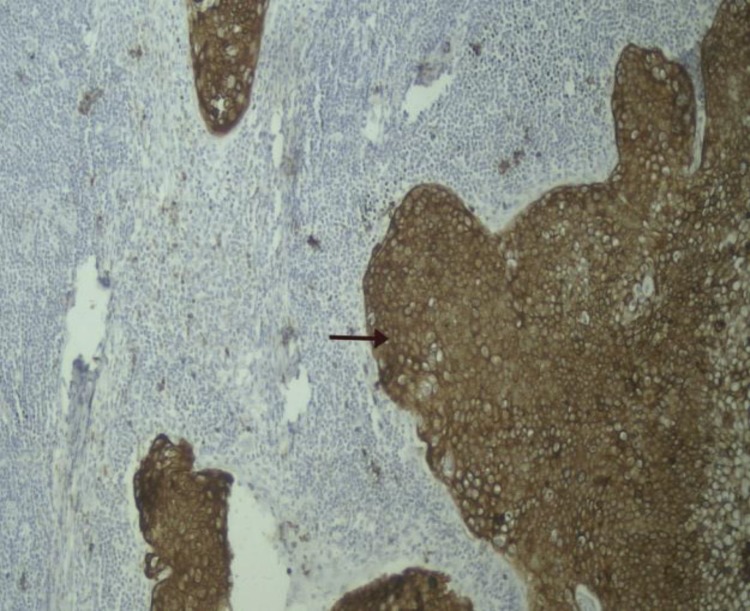
Severe staining for COX-2 in a metastatic lymph node at X100 magnification

**Figure 6:**
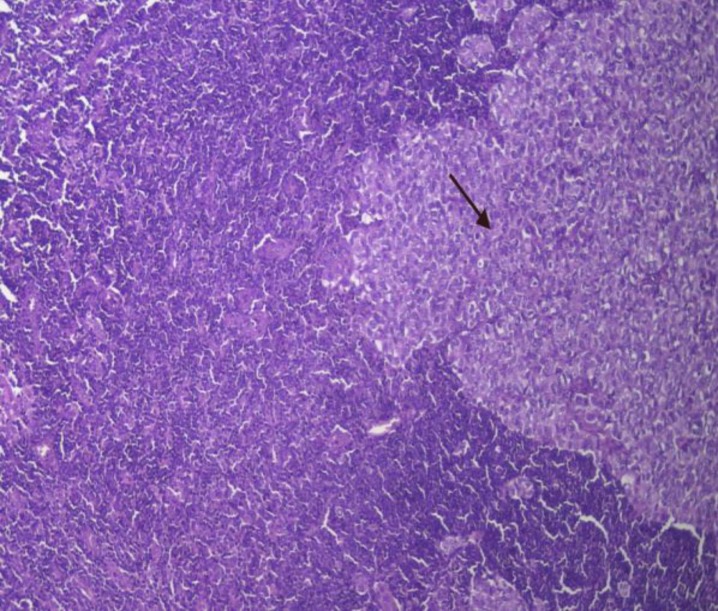
Metastatic melanoma to a lymph node at X100 magnification

Among 23 metastatic melanomas, 16 cases were within lymph nodes, while liver and lung metastases accounted for 4 and 3 cases respectively. A comparison of metastatic and primary melanomas had revealed that COX-2’s expression level had increased during the progression of the disease; a significant correlation was found between COX-2 expression and metastasis (*P*<0.001) (Figure 5,6).

## Discussion

Several studies have illustrated elevated levels of COX-2 in various types of human cancers, including malignant melanoma. Recently, it was reported that COX-2 is strongly expressed in malignant melanoma, and may be correlated with disease development and progression. In contrast, other studies did not detect this protein in primary melanoma cells, but it was found in infiltrating inflammatory or metastatic cells (20-22).

 In Becker's study, COX-2 protein expression was found in 95.0% (96 of 101) of all melanomas; only four melanomas (one spitzoid malignant melanoma, two lentigo maligna melanomas, and one desmoplastic malignant melanoma) showed negative staining results. They found that COX-2 protein expression in melanomas occurs in different SI scores. They evaluated the results based on the four-point scaling system (like our study): no (0 or –), weak (1 or +), distinct (2 or + +), or strong staining (3 or + + +). Strong staining was found in 60.4% (61 of 101) of all melanomas, moderate staining in 22.8% (23 of 101), weak staining in 11.8% (12 of 101), and only five melanomas showed no staining. Furthermore, they employed two types of scoring systems: a manual scoring of the observers and a digital image analyzing system; they found a significant correlation between them (23). In our study, the expression of the COX-2 protein was determined by immunohistochemistry. In 60 cases of primary skin melanomas and 10 benign nevi, we showed that the COX-2 protein was expressed in 59 cases (98.3%). Benign nevi were negative in all cases, so were normal epithelia, and only one Lentigo maligna melanoma showed a negative staining result (1.6%). 

According to different SI scores, strong staining was observed in 60% (36 of 60) of all melanomas, moderate staining in 26.8% (16 of 60), weak staining in 11.6% (8of 60), and only one melanoma (lentigo maligna melanoma) showed no staining; our results were similar to Becker's study. Therefore, it is revealed that the COX-2 protein is strongly expressed in the majority of melanomas.

In another study conducted by Denkert et. al., 28 cases of primary melanoma and 4 benign nevi were analyzed. They reported COX-2 expression in 26 cases (93%) of melanoma, with moderate to strong expression in 19 cases (68%) (18). A greater COX-2 expression was reported in our study in comparison with Denkert's study. They reported that in nodular melanoma, there was a considerable heterogeneity of COX-2 protein expression, with enhancement in the periphery of the tumor. The percentage of cases with strong expression of the COX-2 protein was higher in those with vertical growth (5 cases, 31%) rather than cases with radial growth patterns (1 case, 8%). However, this relationship was not statistically significant. No correlation between tumor thickness and COX-2 protein expression in the tumor, nor in stromal cells, was observed, and no significant differences between COX-2 protein expression and different stages of malignant melanoma were found. There was, however, a reported the expression of COX-2 mRNA and proteins in 5 melanoma cell lines (24). In our study, we observed a significant relationship between tumor thickness and COX-2 protein expression; the latter was also statistically significant in different types of malignant melanoma. We observed strong staining of COX-2 expression in the nodular type of melanoma with 89%, moderate staining in other types of melanoma, mild staining in the superficial type, and negative staining in lentigo maligna melanoma. Most of our patients were categorized as having nodular melanoma and the most expression of the COX-2 protein was seen in this type.

In another study by Meyer et. al., a significant association was reported between advanced Clark levels (*P*=0.004) and shorter recurrence-free survival (*P*=0.03). They suggested COX-2 as a prognostic histological marker in primary malignant melanoma (25). We have demonstrated that the COX-2 protein is strongly expressed in the majority of primary malignant melanomas and is related to the number of mitoses (*P*<0.001), tumor thickness (based on the Clark level and Breslow) (*P*<0.001), melanoma subtypes (0.01), lymph node involvement (*P*<0.001) and metastases (*P*<0.001). These parameters significantly correlate with the SI. Melanomas with a high level of Clark or Breslow are associated with increased chance of metastasis and lymph node involvement. Moreover, higher SI score is predicted with an increase in the level of Clark or Breslow tumor thickness (TT). Therefore, SI scoring can be used as a valuable prognostic marker in all stages of primary cutaneous malignant melanoma. There was understandable disagreement between diagnostic approaches that assess SI scoring. Cases with mild staining had less than 20 mitoses per/10 HPF and cases with moderate to severe staining had more than 20 mitoses per/10 HPF. It is predicted the elimination of other factors is associated with a higher number of mitoses (>20 mitoses per/10 HPF), which is correlated with higher levels of SI scoring. Examination of this hypothesis requires further investigation. 

According to the results of the correlation between the SI scoring system and Clark level, a notably significant difference was seen in the SI of COX-2 expression between Clark levels II and III of melanomas. This correlation might be applicable so as to distinguish melanomas at an early stage of Clark level II from melanomas with higher thickness (Clark level III) to ensure appropriate aftercare. These results emphasize the value of COX-2 as a prognostic marker and a probable therapeutic target in primary melanoma and metastases. In this study, no significant association was found between SI scoring and survival or gender, but further studies with larger sample size and variable melanoma subtypes are recommended. 

## Conclusion

We have demonstrated that COX-2 is strongly expressed in the majority of malignant melanomas and that the SI score of COX-2 is related to the number of mitoses, tumor thickness (based on Clark level and Breslow), melanoma sub-type, lymph node involvement and metastases. No association was noted between anatomic site, gender, and survival. It is concluded that poor outcome (metastasis and lymph node involvement) is related to strong SI staining and high Clark levels.

 The SI scoring system can be used to distinguish benign nevi from malignant melanomas, and COX-2 can be applied as a prognostic factor in malignant melanoma, and a promising candidate for future target therapies. 

Furthermore, we tried to use logistic regression, but it was not possible due to the low number of cases and variable diversity. Therefore, it is advisable to collect more cases with more different subtypes in order to enable the use of logistic regression for data analysis. 

We suggest COX-2 as a valuable immunohistochemical prognostic marker throughout all stages of cutaneous malignant melanoma. Fast Red immunohistochemical staining is suggested for better differentiation of COX-2 protein from the brown-colored melanin. Current and future studies are going to evaluate the effects of COX-2 inhibitors as an effective single or combination therapy against various types of human cancers including malignant melanoma.
